# Can Motivational Interviewing Make a Difference in Supporting Employees to Deal with Elevated Blood Pressure? A Feasibility Study at the Workplace

**DOI:** 10.3390/ijerph18084179

**Published:** 2021-04-15

**Authors:** Martina Michaelis, Carmen Witte (née Farian), Barbara Schüle, Katrin Frick, Monika A. Rieger

**Affiliations:** 1 Institute of Occupational and Social Medicine and Health Services Research, University Hospital of Tübingen, 72074 Tübingen, Germany; carmen.witte@posteo.de (C.W.); monika.rieger@med.uni-tuebingen.de (M.A.R.); 2 Research Centre for Occupational and Social Medicine (FFAS), 79098 Freiburg, Germany; 3 Occupational Health Service, Daimler AG, 70546 Stuttgart, Germany; barbara.schuele@daimler.com; 4 German Academy for Psychology, 10179 Berlin, Germany; info@psycho-login.eu

**Keywords:** arterial hypertension, motivational interviewing, behavioral change counseling, occupational health service, health coach, feasibility study

## Abstract

*Background*: To overcome the problem of a high prevalence of undiscovered or untreated arterial hypertension in people of working age, the effects of behavioral change counseling in occupational health (OH) services should be investigated. The technique of motivational interviewing (MI) to support health-related lifestyle changes by physicians and/or occupational nurses (‘health coach’) has been shown to be successful in patients with chronic diseases. In 2010, we planned a randomized controlled trial (RCT) with employees who suffer from mild arterial hypertension. A preliminary feasibility study was performed in a large manufacturing company in Germany. *Methods*: All employees with elevated blood pressure measured by the OH-service were invited to undergo validation by 30 self-measurements. Persons with validated elevated values and without medical treatment received either usual hypertension counseling (control group, CG) or intensified MI-counseling (intervention group, IG) by the occupational health physician. Subsequently, the IG received MI-support from the ‘health coach’ in four telephone counseling sessions. Assessed feasibility factors included organizational processes, the acceptance of the validation procedure and the MI-counseling, and as primary outcome for an RCT the extent to which participants made health-related changes to their lifestyles. *Results*: Initially, 299 individuals were included in Study Part A (screening). At the end of Study Part B (intervention), out of 34 participants with validated and non-treated mild hypertension, only 7 (IG) and 6 (CG) participants completed the intervention including documentation. The high drop-out rate was due to the frequent lack of willingness to perform the 30 self-measurements at home with their own equipment. Acceptance was little higher when we changed the method to two repeated measurements in the OH service. MI-counseling, especially by the health coach, was evaluated positively. *Conclusions*: Despite the promising counseling approach, the feasibility study showed that an RCT with previous screening in the operational setting can only be implemented with high financial and personnel effort to reach an appropriate number of subjects. This substantial result could only be achieved through this comprehensive feasibility study, which investigated all aspects of the planned future RCT.

## 1. Introduction

Arterial hypertension is a significant risk factor for cardiovascular diseases, especially for strokes and heart attacks, and thus a major health problem in the population. In Germany, between one quarter and one third has known medically diagnosed high blood pressure [1,2,3]. The high prevalence of undetected arterial hypertension [4,5], especially in younger men [6], is a health care problem. Screening studies in different workplace environments and countries identified a prevalence of arterial hypertension ranging between 10% and 40% [7,8,9,10,11,12]. In the German car industry this was even as high as 75% (men) and 47% (women) [13].

Further, rates of unawareness of the condition were, for example, 24% in Canada and 44% in Hungary [9]. In a French workplace study, this concerned almost half of the men and three quarters of the women diagnosed with hypertension [10]. In a younger German study, 13% of *n* = 67 subjects were unaware of their condition [11].

Facing the forthcoming demographic development, it is to be expected that employees must remain efficient and healthy even longer than before to remain a necessary link in the chain of employability [14]. This also places an increasing responsibility on employers and their occupational health services to intensify workplace health promotion activities for aging workers. In addition, employees appreciate an employers’ commitment to maintaining their health [15].

Besides primary care services, occupational health (OH) services are ideal for screening for such conditions, as they come into contact with clients during regular check-up consultations or acute care situations who normally may not have regular contact with a health care provider, e.g., younger men.

Further, support in implementing health beneficial lifestyles is one of the pillars of the WHO Healthy Workplace Model [16,17]. While implementing screening measures in the daily routine of German occupational health physicians seems possible without major effort [18,19,20,21], in Germany, only few intervention studies have been carried out in the occupational setting so far [12,18,20,22,23,24].

Mild arterial hypertension does not necessarily require medical treatment; it is amendable to lifestyle interventions, such as increased physical activity and weight reduction, improved nutrition and moderation of alcohol consumption, salt restriction and smoking cessation [25]. On the other hand, adhering to regular blood pressure measuring protocols as well as implementing and maintaining lifestyle changes are easier to achieve with further support [26,27].

Numerous possibilities have been studied to achieve these changes, as the American Heart Association summarized in a scientific statement: Incentives or motivational interviewing as evidence-based methods can support patients in implementing physical activity and dietary lifestyle changes, in addition to other options, such as goal setting, self-monitoring, and prolonged contact between patients and healthcare professionals [28].

Motivational interviewing (MI) as an approach of behavioral change counseling [29,30] was first developed for addiction counseling [31]. Since the 1990s, MI has also been used in the treatment of chronic diseases, such as cardiovascular diseases, overweight, hypertension, or diabetes mellitus [32,33]. The goal of the directive client-centered counseling approach is to support behavior change in people who feel ambivalent about changing certain behaviors [34] and to build intrinsic motivation to change one’s own behavior. This should be achieved by exploring and resolving ambivalence. Ambivalence is expressed by clients’ statements for or against change: ‘change talk’ or ‘sustain talk’. Client speech predicts client outcome and is associated with MI-adherent or non-adherent counseling behavior [35]. MI has also been used in different settings, such as hospitals [36,37] and primary care for conditions such as chronic pain management, physical activity, and obesity management [38].

Studies evaluating lifestyle counseling in the occupational setting were rare when we initiated our study [22,39,40,41,42,43,44]. However, study designs differed in two RCTs, two cluster-RCTs, one CT, and two observational studies (non-(R)CTs): Either only physicians were involved in lifestyle counseling, but not health coaches [22,40,41], or the MI concept was not always involved [22]. The number of consultations was also not always fixed, but, e.g., depended on the assessment of the attending physician [40]. In addition, there was not always comprehensive health-related lifestyle counseling, but rather a focus on single health problems, such as alcohol addiction [41,42,44]. The health coach concept was pursued in four studies. All of them offered counseling by external protagonists [39,42,43,44], which was considered feasible and successful.

Up to now, evidence of the effectiveness of counseling to change health-related behavior has increased. This concerns individuals with cardiovascular risks [45,46,47], work-related stress [48], musculoskeletal disorders [49], or with diverse other noticeable health-related risks [50,51].

MI has not only been delivered successfully in face-to-face settings, but also as a telephone-based counseling approach [52,53,54]. MI can be delivered by trained personnel from a variety of professional backgrounds [33]. Good experiences were reported in studies in which medical assistant staff counseled patients at risk for cardiovascular diseases [55,56,57]. Three studies found in the occupational health service setting [46,47,51] showed *partial* effects on lifestyle changes.

Based on the positive experiences of the studies mentioned above, it can be assumed that support for lifestyle changes also works for employees with mild arterial hypertension when MI counseling techniques are implemented by occupational health physicians and subsequently by a so-called ‘health coach’ as an alternative to immediate drug treatment. Against this background, the authors planned a feasibility study starting in the year 2010 prior to a future randomized controlled trial (RCT) with the primary outcome ‘extent to which health-related lifestyle has changed’ in terms of eating habits and/or sporting activities.

A single measurement is considered insufficient to identify persons whose blood pressure values are not only temporarily elevated, and might be the result of a so-called ‘white coat effect’ [58]. Thus, the initial value must be confirmed. The gold standard recommendation is a 24-h long-term measurement [59]. However, this procedure is not suitable for the operational setting, given the expected demand for measuring devices. Alternatively, the Austrian Society for Hypertension recommends 30 home self-measurements, of which at least seven must be elevated to allow for a corresponding diagnosis [60]. However, there is no internationally recognized gold standard for home blood pressure measurements [61].

The goal of this feasibility study was to evaluate important parameters such as acceptability and practicable methods of blood pressure measurements, promoting and hindering factors at the level of subjects and organization, and the acceptance of the MI intervention indicated by the number of willing participants to estimate likely recruitment rates of subjects and response rates to follow-up. Here we present the feasibility study and discuss the most important results.

Following the relevant recommendations for complex interventions [62,63,64], we investigated the following research questions:

1. How many employees with so far unidentified or uncontrolled hypertension who have not yet started medical treatment can be identified in the occupational health service of a large manufacturing company as a database for further interventions?
2. How many of the identified employees can be motivated to undergo validation after positive screening for elevated blood pressure? How many of them will perform further self-assessments to validate their initially elevated blood pressure value? What are promoting and hindering factors?
3. How many employees with validated elevated blood pressure can be motivated to undergo hypertension prevention counseling in the occupational health service? How many are willing to be randomized? How many are willing to be subsequently supported by the health coach, and at least how many complete the intervention?
4. Which factors inhibit or support the implementation of motivational interviewing in the occupational health setting?
5. Are the evaluation instruments feasible? How many participants complete all questionnaires up to the end of the study?
6. How do German occupational health physicians and study participants assess the motivational interviewing approach? When we designed the study in 2010, to our knowledge, motivational interviewing had not been used regularly in the occupational health sector to affect employees’ behavior in Germany, with the exception of an alcohol use intervention program.

The study was approved by the ethics commission of the medical faculty of the Eberhard Karls University of Tübingen (329/2010BO1). This manuscript follows the recommendations for reporting on feasibility studies of the CONSORT group [65].

## 2. Materials and Methods

### 2.1. Study Part A (Methods of Screening and Validating Hypertension)

All employees who, planned or in the event of an emergency, visited the occupational health service between October 2010 and April 2011 underwent a blood pressure measurement using the Riva-Rocci (RR) method. Recruiting staff were physician assistants, laboratory personnel and staff of the emergency department.

After informed consent, employees with values between 140/90 and 159/99 mmHg were asked to take part in Study Part A with 30 repeated domestic self-measurements with their own equipment. The equipment could be obtained at a reduced price from the occupational health service (OHS), if necessary, and were required to have a seal of approval from the German High Pressure League (*Deutsche Hochdruckliga*) [66].

We calculated 50 persons with complete follow-up data for intervention and control groups, respectively. During previous OH service screenings, an average of seven employees with elevated blood pressure values between 140/90 and 159/99 mmHG (8% on all examination occasions) were found per working day, mostly subjects without medical treatment. Under the (unfavorable) assumption that only a quarter of the employees could be recruited to participate in a fully completed study, four recruitment months were calculated.

To increase the return rate of the completed documentation, a maximum of three telephone reminder attempts were made after the agreed return period was exceeded. Recruiting for this kind of validation started in February and ended in April 2011.

Due to the low willingness of the employees to participate in this kind of self-measurement (see results section), we changed the validation procedure to two repeated measurements in the OH service between February and April 2011. A median of ≥140/90 mmHg of the total of three values confirmed the suspected arterial hypertension. This fulfilled the requirements documented in guidelines for repeated monitoring of elevated blood pressure values to confirm the diagnosis. However, there is no indication in available studies that two repeated blood pressure measurements are sufficient to confirm the diagnosis of arterial hypertension. We were aware of that, yet we considered this a feasible procedure for the OH setting.

The blood pressure values measured at home were documented by the study participants themselves using a ‘blood pressure card’. The values measured in the OH service were documented similarly by the staff. The documents for the self-measurements were handed out in the OH service and sent back through the internal postal service of the enterprise using closed envelopes to secure confidentiality.

If the values continued to be slightly elevated between 140/90 and 159/99 mmHg, the respective employees received an invitation to an individual hypertension prevention counseling. Exclusion criteria for participation can be found in Online Supplementary Table S1.

For ethical reasons, usual care counseling was also offered to employees who were not invited to the intervention due to exclusion criteria. Employees with highly elevated blood pressure (≥160/100 mmHG) were motivated to visit their primary care physician or a medical specialist. Further developments and results of the adherence to this recommendation were evaluated separately as part of a medical doctoral thesis [67].

### 2.2. Study Part B (Intervention Methods)

Employees with verified hypertension values were informed about the results of the repeated blood pressure measurements by at least three telephone attempts and in the case of unavailability by a subsequent letter. If the inclusion criteria were met (slightly elevated blood pressure between 140/90 and 159/99 mmHg), they were invited to participate in the study and, if interested, were given an appointment for counseling with an OH physician. Even if the employees were not interested, they were offered usual care hypertension counseling by another company physician.

Shortly before counseling, study participants were given detailed information at the OHS. After giving informed consent, participants were randomly assigned to either the intervention group (MI-counseling by an occupational health physician (OHP) at time T0 and subsequently by the health coach) or the control group (usual care counseling by an OHP) using single blinded block randomization (block size *n* = 4, freeware tool Research Randomizer).

Four physicians and the health coach were trained by an expert in behavioral change counseling using MI during an interactive one-day workshop. The basic documents for consultation and documentation were developed with the support of another expert (KF) [68,69].

**Figure 1:**
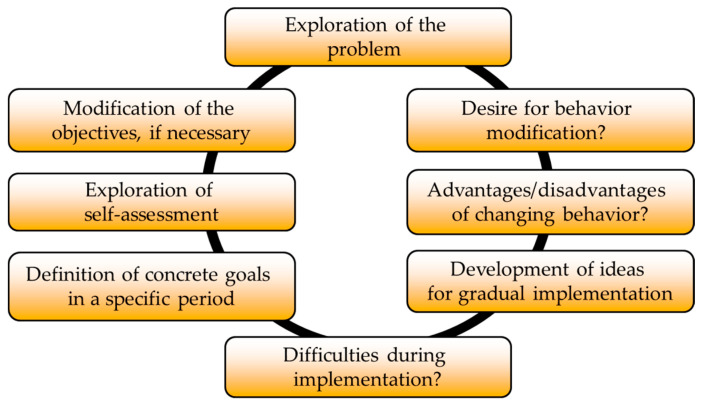
Procedure and content of motivational interviewing (MI)-counseling in the intervention group. Legend: Representation based on Information from [68] and adapted from [70].

The objectives of the health-related lifestyle counseling for the intervention group were an evidence-based expansion of health-related knowledge and the ability to self-manage based on the MI-principles and attitude (‘MI-spirit’). These principles are empathy and respect for the autonomy of the patient/volunteer, enhancing intrinsic motivation, focusing on the client’s goals, developing discrepancy between clients’ goals or values and their current behavior, and exploring ambivalence, as well as supporting self-efficacy. Reflective listening helps to recognize and evoke ‘change-talk’. Using MI-methods, individual health goals were developed mainly from the subject areas of nutrition and physical exercises (see process shown in Figure 1).

The intervention group was informed by the MI-trained occupational health physician about risk factors for developing arterial hypertension and possible lifestyle changes regarding a healthier diet, more exercise, (better) stress management strategies, but also abstaining from smoking. The so-called Score Germany table was used to illustrate the overall cardiovascular risk and possible effects of behavioral changes [71].

The control group received usual care in the form of less intensive counseling by the same four occupational health physicians. The MI-related documents developed by our group were used as the basis for counseling the intervention group, and the standard OH service documents were used for counseling the control group.

Finally, both groups received a hypertension prevention brochure designed by their employers’ workplace health promotion department and were informed in detail about in-house and external health promotion offers and motivated to participate.

Subsequently, an MI-trained medical student (CW) acted as health coach for the intervention group and accompanied the implementation of individual health goals established during counseling with the OH physicians into concrete, realistic steps. This was carried out by four counseling sessions via telephone during the employees’ leisure time. Intervals between the contact times T1 through T4 were as follows: T1 = T0 + one week, T2 = T1 + one week, T3 = T2 + three weeks, T4 = T3 + four weeks.

### 2.3. Evaluation Methods and Instruments

The feasibility of both screening and intervention was assessed in a mixed-method design following the dimensions process and outcome evaluation according to Donabedian [72]; see Online Supplementary Tables S2 and S3; all evaluation instruments were developed by the authors. Experiences within the recruiting processes were collected in protocols and semi-standardized group and expert interviews with the OH service staff.

Experience and satisfaction with hypertension prevention counseling in the OH service was rated in a standardized questionnaire by all participants six months after the OH physician consultation at T5 (i.e., T4 + nine weeks). The experience of counseling by the health coach was evaluated only by the intervention group.

To assess lifestyle changes as a primary outcome, a standardized questionnaire was deployed at baseline (T0) and T5 (final evaluation), aiming to compare data on dietary habits, exercise behavior, and nicotine consumption. The assessment of the subjectively rated lifestyle changes at T5 included six items that were combined into a standardized sum score.

Secondary outcomes at T5 were blood pressure values, the general expectation of self-efficacy, self-assessed health, and the importance of own goals, self-confidence, and readiness to work toward these goals. Details of Outcomes and evaluation methods can be derived from Online Supplementary Tables S2 and S3.

### 2.4. Statistical Analysis

Results from standardized questionnaires are presented descriptively by percentages or mean values/standard deviations (SD)/median. Cross-sectional group differences were analyzed by Chi^2^ and nonparametric Mann–Whitney U tests. Significance threshold value was set to *p* = 0.05 (Study Part A) and *p* = 0.10 (Part B due to low sample sizes). The respective effect sizes correlation coefficient phi and r (z/root(*n*)) were categorized with low (<0.3), moderate (0.3–0.5) and high (>0.5) [73].

For Part A (validation of blood pressure measurements), the examination of individual predictors on the outcome ‘willingness to participate in repeated measurements’ was analyzed exploratively, as the sample was large enough for multivariate logistic regression (the respective effect size was odds ratio with 95% confidence interval). Based on the simultaneous inclusion of all previously bivariate-tested variables, which showed *p* < 0.20 (method = ‘enter’), nine predictor variables were tested (see Online Supplementary Table S4).

## 3. Results

### 3.1. Study Part A (Screening and Validation of Hypertension)

#### 3.1.1. Results of Outcome Evaluation: Number of Individuals with Mild Hypertension

The study period during which validation of the initial measured blood pressure values was performed with 30 self-measurements lasted five months; *n* = 203 followed the invitation for blood pressure validation. This period lasted an additional two months for the subsequent 2RM group (*n* = 96). During that time, a total of 299 employees with initially elevated blood pressure values were identified among *n* = 2181 who took part in the screening. Out of these, 90.3% were men and 66.1% were employed in production or production-related areas. The mean age was 45.3 years (standard deviation SD 9.0).

**Figure 2:**
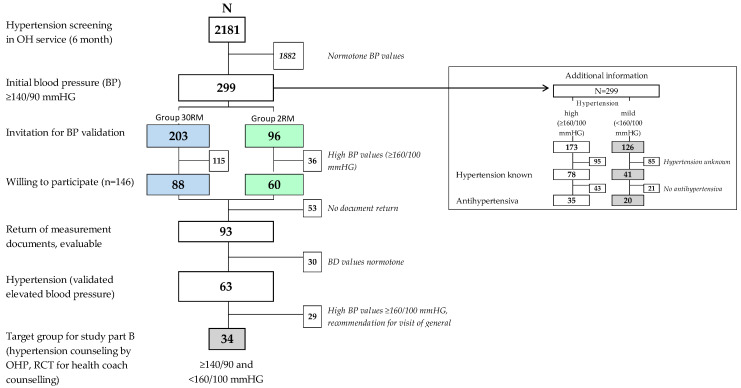
Participants in Study Part A (screening and hypertension validation).Grey shadowed values = employees with slightly elevated blood pressure values (≥140/90 and <160/100 mmHG). Abbreviations: 30 RM = group with 30 repeated self-measurements; 2 RM = group with 2 repeated measurements in the occupational health service; OHP = occupational health physician; OHS = occupational health service.

In total, less than half of the participants were aware of diagnosed hypertension (*n* = 119/299; 39.7%; see additional information in Figure 2). Especially in the high blood pressure group, only one out of five individuals was undergoing medical therapy at the time point of the initial screening (20.2%; *n* = 35 out of 173).

#### 3.1.2. Results of Process Evaluation: Feasibility of Screening with Focus on Employees

The willingness of employees with initially elevated values to participate in repeated blood pressure measurements was slightly lower in group 1 (30 RM) than in group 2 (2 RM) with two repeated measurements in the OH service (43.3% vs. 63.5%; *p* = *0*.001; phi = 0.2; *n* = 88/203 vs. 60/96). The individual results of blood pressure measurements were documented on so-called blood pressure cards, yet these were not returned reliably in either group, with a lower rate in group 1 than in group 2 (49/88 vs. 39/60, not significant). The necessity of telephone reminders to submit results was also significantly greater for group 1 than for group 2 (69.4% vs. 25.6%; *n* = 34/49 vs. *n* = 10/39).

A completed blood-pressure card was evaluable from a total of 88 participants (59.4%) out of the *n* = 148 who agreed to undergo validation (see again Figure 1; no statistical differences between validation groups).

The most important reasons for not participating in the validation measurement procedure were lack of interest, the time required for the procedure, and existing medical treatment. There were no statistical group effects (see Online Supplementary Table S5).

The results of the multivariate regression analysis showed that the willingness to participate was twice as high in group 2 (2 RM) compared to group 1 (30 RM); OR = 2.1, CI_95%_ = 1.2–3.6. Willingness to participate was 50% lower for employees with current antihypertensive medication and for employees working in production compared to the reference group ‘administration’ (both OR = 0.5, CI_95%_ = 0.2–0.9 and 0.3–0.9, respectively).

The lowest participation rate was estimated for employees working in production with previous medical therapy in group 1 (see Online Supplementary Figure S1). All variables were statistically significant in the model except for age, which was slightly below the threshold for significance (*p* = 0.051). No predictive value was found for blood pressure level at the initial measurement, the profession of the recruiting person in OH service for the initial measurement, the reason for visiting the OH service, knowledge of previously diagnosed hypertension, or gender.

#### 3.1.3. Results of Process Evaluation: Feasibility of Screening with Focus on OH Service

In general, all documentation sheets within the study process were completed without gaps and were found to be suitable by OH service personnel.

Detailed results of the group interview with four medical assistants to evaluate the recruiting process are presented in Online Supplementary Table S6. In summary, it was considered possible to integrate the recruitment interviews into the daily work routine, but the high proportion of employees with poor language competence generated problems. In addition, skepticism about data protection issues were reported, as well as reluctance to participate after initial interest due to the effort associated with repeated domestic measurements in the group with 30 repeated blood pressure measurements (30 RM). Improvements proposed for the study process included forgoing the 30 domestic measurements in favor of two repeated measurements in the OH service practice, multilingual study information, and visually attractive brochures.

### 3.2. Study Part B (Intervention)

#### 3.2.1. Feasibility of RCT Design and of Hypertension Counseling by OH Physicians (Focus on Participants)

**Figure 3:**
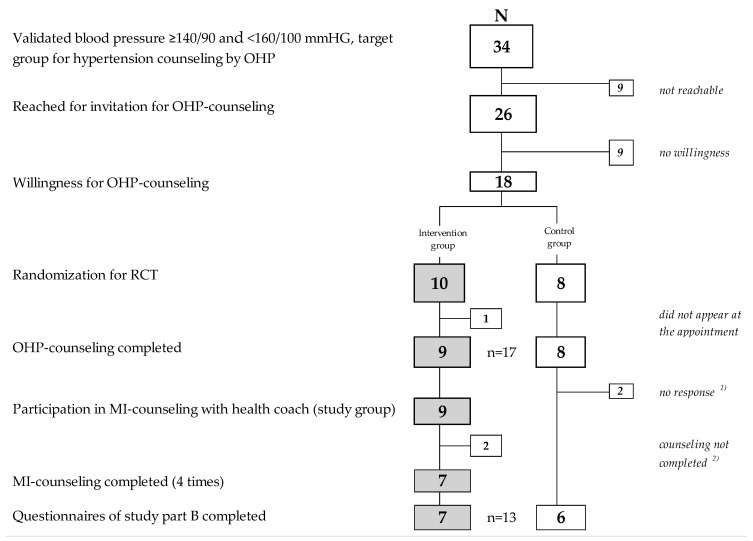
Participants in Study Part B (intervention with randomized controlled trial (RCT) study design). Legend: ^1)^ to complete T5 questionnaire after telephone reminder; ^2)^ not reached any more.

From 62 participants with validated elevated blood pressure (see again Figure 2), a target group of *n* = 35 with mild hypertension (≥140/90 and <160/100 mmHg) remained for randomization, followed by additional counseling by the health coach (IG), or no additional counseling (CG). In total, *n* = 26 were reached for invitation and were randomized prior to the counseling date (see Figure 3). Similar to Study Part A it was time-consuming to reach the employees in shift work to inform them about verified hypertension results and invite them to receive counseling by the OH physician.

*n* = 18 were invited to the health coach counseling and *n* = 13 completed this counseling. The end of Study Part B reached 13 participants (IG: *n* = 7, CG: *n* = 6) out of the target group.

During counseling in the intervention group, the self-assessment of the importance of own goals was high (median 7.8, with a possible range from 1 ‘not at all’ to 10 ‘very much’), self-confidence was medium (median 6.0), and readiness to work for these goals relatively low (median 4.0; cases answering to the three items: *n* = 9/9/7).

IG and CG assessments of MI-counseling by the OHP resulted in mean values of 2.14 (SD 1.17) and 2.38 (SD 1.19; sum score; possible range from 1 ‘fully applies to 5 ‘applies not at all’), respectively. The respective mean satisfaction score was 1.82 (SD 1.20) in IG and 2.54 (SD 1.05) in CG. No statistical differences or positive effect sizes were found (r_sum score_ = 0.05 and 0.03; *p* = 0.534 and 0.295). More details are presented in Online Supplementary Table S7.

#### 3.2.2. Feasibility of Study Processes and OHP Counseling–Assessment of OH Physicians (Focus on Own Work)

The median duration for the behavioral change counseling was 25 min in the intervention group and 17 min for the usual counseling in the control group, respectively. All documentation forms about communicating individual topics in lifestyle counseling were completed without gaps and found to be suitable by the OHPs.

The median sum scores of satisfaction with the MI-method and respective procedures were 2.92 with a range from 1 ‘very satisfied’ to 5 ‘not satisfied at all’ (satisfaction with the concept of motivational interviewing), 2.25 (satisfaction with familiarization with the study design, 2.25 (satisfaction with communication processes in the study team and the complex documentation procedure), but only 4.50 for ‘satisfaction with the integration of MI into the daily work routine’ (see Online Supplementary Table S8).

#### 3.2.3. Feasibility of Repeated MI-Counseling by the Health Coach (Focus on Participants; IG Only)

The telephone calls lasted on average 20 min (range 5 to 40 min). During counseling, the seven participants in the intervention group seemed to be highly motivated. This is also reflected:

- In the 12-item sum score concerning assessment of and satisfaction with the concept (median 3.7, ranging from, 1 ‘fully applies to 5 ‘not applies at all’ (see Online Supplementary Table S9);
- In the oral feedback during the four health coach MI-counseling sessions. The self-assessment of importance of own goals (median 7.3–10.0 from T1 to T4 with a possible range from 1 ‘not at all’ to 10 ‘very much’), self-confidence (median 4.0–8.8), and readiness to work for these goals (median 8.0–10.0).

#### 3.2.4. Intervention Effect on Primary Outcome ‘Lifestyle Changes’

(a) RCT group comparison; *n* = 7 IG, *n* = 6 CG; T5 6 months after baseline evaluation: There was a moderate, but due to the small sample size statistically non-significant improvement in the primary outcome ‘lifestyle changes’ as an overall score of 0 ‘not at all’ to 4 ‘very strong’ compared to the control group (r = 0.49; mean value _(IG)_ = 4.43, SD 0.54, range 4–5; mean value _(CG)_ = 4.00, SD 0.00, range 4–4, median both 4.0).

The three smokers in IG reduced their nicotine consumption slightly with a tendency towards moderate effects compared to four smokers in CG (r = 0.28; mean value (IG) = 2.67 (SD 2.31), mean value (CG) = 1.00 (SD 2.00), median both 4.0, range both 0–4.

#### 3.2.5. Intervention Effect on Secondary Outcomes at T5

At T5, four out of seven persons in IG indicated having achieved their previously defined personal goals.

All six persons in IG and five out of seven persons in CG reported that they continued measuring their blood pressure from time to time at T5. The two other CG subjects stated that they did not have an own measurement device. The blood pressure card was available from *n* = 5 (IG) and *n* = 3 (CG). Due to the small number of cases, we waived the statistical evaluation.

## 4. Discussion

We present results of a feasibility study performed prior to a future randomized-controlled effectiveness study between 2010 and 2011. It was planned to focus on the effect of behavioral change counseling concerning a healthy lifestyle among employees with validated mild hypertension and without previous drug therapy. We used the motivational interviewing method, which is considered to be successful in different patient groups. One of the aims of the feasibility study was to apply the method for secondary prevention in the occupational health service setting with persons who generally are little motivated to visit their general physician for prevention, e.g., due to their gender (men) or age (younger) and thus to their general health status.

The novelty in this study was the counseling provided by a combination of MI-trained occupational health physicians plus a perpetuating MI-trained health coach. As described above, only a few studies could be found when we started our project in 2010. Since then, seven counseling studies have been published which address the occupational setting’s ability to support behavioral lifestyle change, with diverse study designs [45,46,47,48,49,50,51]. In two RCTs, two cluster-RCTs, and three observational studies, counseling was again performed exclusively by physicians, [45,47,49] or by team leaders or other staff [48,50]. Only the study by Groeneveld et al. [46] is more or less comparable to our design, since the counseling was conducted by occupational health physicians *and* an occupational nurse.

Further, newer counseling study designs vary in terms of the intensity of MI-counseling (one-time [50], variable number of consultation units [49], higher intensity compared to our study [46,47]). In addition, only partial MI-elements were used instead of the entire MI-concept [46,50,51], or counseling to support behavioral lifestyle change took place without motivational interviewing techniques [45], or group intervention was used instead of individual counseling [48]. Interventions were related to comprehensive health-related lifestyle aspects to prevent cardiovascular risks [45,46,47,51], motivation to take advantage of well-being programs [50], physical activities and relaxation to reduce stress-related need of recovery [48], or the adherence to therapy recommendations of rehabilitation patients for a faster return to work [49]. None of these studies focused on hypertension as a primary approach. However, positive effects were reported in half of the newer studies, except for no effects in one study [47] and only partial effects in two other studies [46,51].

Our results also show that validating a single elevated blood pressure value using 30 self-measurements instead of the gold standard 24-h continuous measurement was too much effort for our subjects. The participants lost motivation over the four-month period, which subsequently led to a high drop-out rate.

Further, the requirement of participants to finance the measuring device themselves might have caused a selective sample of “willing to pay”. It can be assumed that making measuring devices available free of charge would have led to a higher level of acceptance; however, this would have been beyond the scope of what is feasible for an employer.

Acceptance was increased using a simplified validation procedure; this was reached using only two repeated blood pressure measurements, which is beyond the gold standard for validating an initially elevated blood pressure value. On the other hand, this proved to be a feasible procedure for the OHS setting [70]. The procedure was discussed with Seibt et al., who in the 1990 s identified a minimum of 15 self-measurements on three days as sufficient diagnostic confidence [74]. To rule out false diagnoses effectively, the authors currently recommend repeated self-measurements completed by a 24-h measurement [75], which is even more costly for employees and therefore does not seem feasible for a project in the vocational setting.

Since our feasibility study also showed that many employees with elevated measured values during the screening preferred to consult their general practitioner, consideration should be given to how the hypertension validation can be successful under consideration of general medicine (see the study by Randerath et al. [11]). However, the participation rate in this study was also low.

Regarding study procedures, the screening itself was feasible; potential for improvement was only evident in minor cases. On the other hand, the effort required to complete the survey instruments and telephone contact with employees working in shifts was enormous, which would have to be accounted for in personnel cost planning for an effectiveness study.

Given the small number of cases, the positive effects on the primary outcome ‘lifestyle changes’ in our intervention study (Part B) were not statistically significant. However, associated moderate effect sizes show that the partial effects found in comparable, more recent studies [46,47,51] could also be confirmed with a larger sample.

### Strengths and Limitations of the Study

The strengths of the study lie in the comprehensive assessment of the feasibility of all aspects of a future (cluster-)randomized-controlled effectiveness study. Besides the proof of feasibility and value of MI counseling with small case numbers in the setting of an occupational health service, and implementation of two different regimes for validation of initially elevated blood pressure, one of the main results described the high effort necessary for the future effectiveness study. Chances and limits of a future, as yet unplanned RCT, as well as practical suitability beyond study conditions can be derived from our feasibility study.

Limitations: We refrained from estimating the willingness to participate in the screening part of the study (i.e., validation of the elevated blood pressure reading) in a small sample prior to the presented feasibility study. In addition, the study results were generated in a large company with a centrally located in-house OH service. Transferring the design to companies with an in-house but spatially distant or external OH service seems to be possible only to a limited extent. The participants in the randomized-controlled part of the study comparing both counseling methods may be a positive selection, as only the employees who performed validation of the blood pressure measurement were asked to participate in Part B of the study. A meaningful analysis of intervention effects was not possible due to the small number of intervention cases.

There was no evaluation of MI-adherence of counsellor behavior to assess quality and integrity of the MI-interventions [76].

## 5. Conclusions

To our knowledge, this feasibility study is one of few with a randomized-controlled design that also includes the complete MI concept to counsel employees with an initial chronic health problem by occupational health physicians and a company health coach. Undetected slightly elevated blood pressure is symptom-free and thus the level of suffering and the subjective need for lifestyle behavior change is low. Therefore, we conclude that the intended optimization of health in younger employees of our study design has an unbalanced cost-benefit ratio.

Even though the motivation of those who could be recruited for the intervention was recognizably high, an effectiveness study (i.e., an RCT or cRCT) according to the described design would be feasible, but expensive and time consuming. Further, the high effort in recruiting affected persons appears not to be practicable beyond study conditions.

Further, following the results of Jarbøl et al., people with health problems seem to change their lifestyle rather than take medication to reduce cardiovascular risks—at least intentional [77]. Therefore, the importance of counseling by the occupational health service to avoid harmful behavior should not be underestimated. This would allow a long-term relationship to be built up with young, high-risk individuals who are not yet suffering from health problems, and the motivation for health-related changes in behavior can be worked on.

Future RCTs should assess MI-adherence of counseling behavior.

## Data Availability

The data presented in this study are available on request from the corresponding author.

## Supplementary Materials

The following are available online at https://www.mdpi.com/article/10.3390/ijerph18084179/s1, Online Supplementary Table S1: Exclusion criteria for participation in study part A (blood pressure screening), Table S2: Outcomes and evaluation methods in Study Part A (screening), Table S3: Outcomes and evaluation methods in Study Part B (intervention), Table S4: Predictor variables on the outcome ‘willingness to participate in repeated measurements’ (study part A), Table S5: Reasons for non-participation by type of validation measurement—results of recording at first contact in a semi-standardized process protocol (multiple answers), Table S6: Answers of medical assistant staff concerning experiences recruiting employees for participation in study part A (screening); group interview (4 out of 5 persons; multiple answers possible), Table S7: Occupational health physicians’ hypertension prevention counseling –assessment and satisfaction of participants of intervention and control group, Table S8: Satisfaction with the MI-method and respective procedures from the point of view of the occupational health physicians (*n* = 4), Table S9: Experiences with the telephone MI-counseling by the health coach (intervention group), Figure S1: Participation in hypertension validation by work area, treatment status, and validation type.
