# Supplementary material for: Can Motivational Interviewing Make a Difference in Supporting Employees to Deal with Elevated Blood Pressure? A Feasibility Study at the Workplace
Source: Int J Environ Res Public Health. 2021 Apr 15;18(8):4179. doi: 10.3390/ijerph18084179 (PMC8071336; doi:10.3390/ijerph18084179)
Supplement: Supplementary file 1 [file ijerph-18-04179-s001.zip › ijerph-1157539-supplementary material.pdf]

Online Supplementary

Can motivational interviewing make a difference in aiding employees to deal with elevated blood pressure? A feasibility study at the workplace

Table S1. Exclusion criteria for participation in study part A (blood pressure screening).

Strongly increased values (≥ 160/100 mmHg)
Previous hypertension drug treatment
Previous diseases and other medical reasons for secondary hypertension (kidney diseases, hormonal disorders, such as hyperthyroidism or adrenal dysfunction), diseases of the central nervous system (inflammation, increase in intracranial pressure, stroke, medical history of myocardial infarction, arterial occlusive disease/claudicatio intermittens)
Pregnancy
Drugs such as appetite suppressants or non-steroidal anti-inflammatory drugs in continuous use >6 weeks
Age younger than 18 or older than 64
Little knowledge of the German language

Table S2. Outcomes and evaluation methods in Study Part A (screening)

**Outcomes**		**Methods (instruments)**
**Process evaluation**	Feasibility of screening (focus on employees)	Process protocol: Receipt of a completed blood pressure card unsolicited or after a telephone (max. 3 attempts) / written reminder? Participant reachable by telephone? Willingness to participate maintained? Completed blood pressure card (quality of the entries) evaluable? Drop-outs: Reasons?
	Feasibility of screening (focus on occupational health service)	Semi-standardized group interview with the medical assistant staff based on six key questions: Possible integration of the recruitment interviews into the daily work routine, reaction of the employees regarding the topic of hypertension and the study, best motivation for participation, reasons for a refusal, possibilities for optimizing the recruitment process?
**Outcome evaluation**	Number of individuals with mild hypertension (140/90-159/99 mmHg); primary outcome	Results of blood pressure card: [2)] Group '30RM': Median of all elevated values ≥140 resp. ≥90 mmHG out of 30 measurements (at least 7 values must be elevated) [**Error! Reference source not found.**]<br>Group '2RM': Median of all three values (one initial and two repeated measurements)

Table S3. Outcomes and evaluation methods in Study Part B (intervention)

Outcomes [1)]		Methods (instruments)
**Process evaluation**	Feasibility of RCT design	Number of individuals participating in an RCT intervention study
	Feasibility of hypertension counseling by OH physicians in both IC and CG (focus on study participants)	Process protocol: Can persons be contacted by telephone to report medical findings and to be invited to counseling by an OH physician? Do they keep the date? If not, can they be contacted again by telephone?
	Feasibility of MI-counseling. Assessment by OH physicians (focus on OH physicians)	Semi-standardized questionnaire answered during expert interview with the OH physicians; 12 items covering four dimensions of satisfaction with<br>1. the concept of motivational interviewing (MI),<br>2. the integration of MI into daily work routines,<br>3. the introduction to become familiar with the study design,<br>4. communication processes in the study team and the complex documentation procedure.<br>Likert-scaled between 1 'fully applicable 'to 5 'not applicable at all'.<br>Reversal of polarity of negatively formulated items. Construction of mean scores described by mean, standard deviation, median. Likert-scaled between 1 'fully satisfied 'to 5 'not satisfied at all
	Feasibility of repeated MI-counseling by health coach and self-management of the participants	Process protocol: Can participants be reached by telephone? Willingness to participate in telephone MI- counseling ensured? If no, reasons? Willingness to repeat three blood pressure measurements in the OH service? If no, reasons? Follow-up blood pressure card completed and received in a collecting box?
**Outcome evaluation**	Lifestyle changes [3)] comparing a) results of T0 and T5 [4)] and b) subjectively rated by participants at T5; primary outcome	a) Block Food Frequency Questionnaire BFFQ [1]; Freiburg and Questionnaire on Physical Activity FFKA [2];<br>b) Six items (increase in physical activity, change of diet (less/healthier food), reduction of alcohol, better handling of stress, reduction/ cessation of nicotine consumption, and other health-promoting behavioral changes); Likert-scaled between 0 'not at all' and 4 'very strong'
	Assessment of and satisfaction with hypertension prevention counseling by OH physicians (participant's point of view) at T5; secondary outcome	Standardized questionnaire for IG and CG, Likert-scaled answers:<br>• Assessment of counseling (four items): It helped me to be clear about my health behavior; the expectations from it have been fulfilled; I would recommend it to a friend/colleague; it was an important trigger to want to change my health behavior (range from 1 'fully applicable' to 5 'not applies at all').<br>• Satisfaction with… (four items) the information I received; the feedback and advice I received; the way the OH physicians conducted the counseling; the way the OH physicians addressed my personal situation (range from 1 'very' to 5 'not satisfied at all).
	Experiences with telephone MI- counseling by health coach (participant's point of view) at T5; secondary outcome	Standardized questionnaire (IG only):<br>• 12 items including one global satisfaction item ranging from 1 'fully applicable' to 5 'not applicable at all'): I was able to integrate the telephone calls well into my daily routine; the atmosphere in the conversation was positive/pleasant; I felt understood and that my views were taken seriously; I was listened to attentively during the conversation; I was encouraged to talk about my current health behaviors; my choices and achievements so far were acknowledged during the conversation; I was able to achieve agreed-upon goals at my own pace; the phone calls were an important trigger to want to change my health behaviors; I would recommend

**Outcomes** [1)]		**Methods (instruments)**
		the phone calls to a friend/colleague; the phone calls were very helpful in keeping me "on track" with my health goals; the expectations I had for the phone calls were met; overall, I am very satisfied with the telephone consultations. Sum score construction of three dimensions (satisfaction with MI- concept, with integration of MI into the daily work routine, and with introduction into the study) described by mean, standard deviation, median)
	Reported self-assessment of achievement of previously defined personal goals of lifestyle change; secondary outcome	Part of MI-counseling, oral self-assessments documented by OH physician and in the same way repeatedly during every health coach contact: Importance of own goals, self-confidence, and readiness to work toward these goals. Likert-scaled answers from 1 'not at all' to 10 'very much'
	Blood pressure values at T5; secondary outcome	Follow up blood pressure card (three measurements in the OH service): Median of all measured values

Legend:

Note: All evaluation instruments were developed by the researchers in the study team, with the exception of lifestyle change questionnaires.

Abbreviations: CG= control group; IG= intervention group; MI = motivational interviewing; OPH=occupational health physician

[1)] Intervention for persons with identified mild hypertension

[2)] Measurement group '30RM' (30 repeated (self-)measurements, group '2RM': two repeated measurements after the initial measurement in the OH service

[3)] Dietary habits, exercise behavior and nicotine consumption

[4)] Comparing results of T0 (baseline evaluation) and T5 (final evaluation, six months after the OH physician consultation)

[1] Block, G. Improving diet methods, improving epidemiologic methods. Ann. Epidemiol. 1994, 4, 257–258. German version in Suter, P.M. Checklist Nutrition. (In German: Checkliste Ernährung), 3rd ed., Thieme: Stuttgart, Germany, 2008.

[2] Frey, I.; Berg, A.; Grathwohl, D.; Keul, J. Freiburg Questionnaire of Physical Activity—development, evaluation and application (in German: Freiburger Fragebogen zur körperlichen Aktivität-Entwicklung, Prüfung und Anwendung). Soz. Praventivmed. 1999, 44, 55–64, doi:10.1007/BF01667127.

Figure S1. Participation in hypertension validation by work area, treatment status, and validation type.

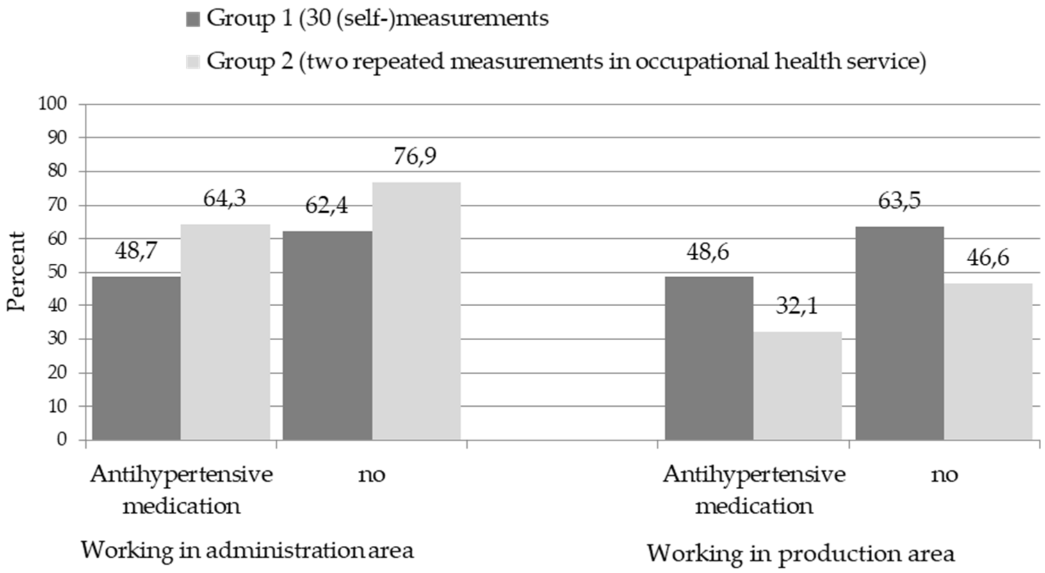

Legend: Estimated values derived from logistic regression analysis (n=249 out of 299); model parameters: $Chi^2_{(model)}$ = 43.9, df=4, $R^2_{(Nagelkerke)}$ = 0.09, correct classification of cases = 63.1%, $-2LL_{(model)}$ = 157.9. Variables excluded from the final model: Age (missed the significant threshold with p = .051), blood pressure level at the initial measurement, profession of the recruiting person in the OH service for the initial measurement (physician assistants or laboratory personnel), reason for visiting the OH service, knowledge of previously diagnosed hypertension, and gender.

Table S4. Predictor variables on the outcome 'willingness to participate in repeated measurements' (study part A)

Type of validation measurements (group 1: 30 repeated (self-)measurements ('30RM') vs. group 2: two repeated measurements in the OH service ('2RM');
Blood pressure level at initial measurement (group 1: ≥140/90 to 159/99 or group 2: ≥160/100 mmHG);
Recruiting person in OH service for the initial measurement (physician assistants/ laboratory personnel vs. staff of the emergency department);
Reason for visiting the occupational health service (occupational health examination vs. other);
Knowledge of previously diagnosed hypertension (yes/ no);
Hypertension medication status (yes/ no);
Work area (administration vs. production/ production-related);
Age
Gender

Table S5. Reasons for non-participation by type of validation measurement – results of recording at first contact in a semi-standardized process protocol (multiple answers)

	Percent (n 'yes')	
	**Group 1 (30RM) n=97**	**Group 2 (2RM) n=34**
Not interested without further reason [1) 2)]	24.7 (24)	29.4 (10)
No time [1) 2)]	29.9 (29)	23.5 (8)
(Pre-)treatment by general physician/ medical specialist [2)]	32.0 (31)	15.5 (15)
No measurement device available at home	10.3 (10)	0.0 (0)
Blood pressure control desired by general care physician	7.2 (7)	0.0 (0)
No willingness to acquire measuring device	7.2 (7)	0.0 (0)
Not convinced of the importance of the measurements [1)]	5.2 (5)	8.8 (3)
Data protection reasons [1)]	2.1 (2)	0.0 (0)
Insufficient language skills [1)]	2.1 (2)	0.0 (0)
Not eligible for Study Part A [3)]	2.1 (2)	2.9 (1)
Blood pressure otherwise within normal range	3.1 (3)	0.0 (0)
Overload/stress/too much effort	2.1 (1)	0.0 (0)
Time for consideration desired	2.1 (2)	2.9 (1)

Legend: N = 131 respondents out of 151 non-participants (group 1: n=97, group 2: n=34); base of percentages is number of cases; answers sorted by percentages in group 1.

Abbreviations: 30RM = 30 (self-)measurements, 2RM= two repeated measurements in the occupational health service practice.

Footnotes: [1)] standardized responses, others summarized into categories; [2)] no significant group difference (p>.05); [3)] because of e.g. vacation, other medication, hospital stay

Table S6. Answers of medical assistant staff concerning experiences recruiting employees for participation in study part A (screening); group interview (4 out of 5 persons; multiple answers possible).

Question	Answers
1. How well can recruitment interviews be integrated into the work routine?	• Recruitment interviews are easy to integrate, e.g. during blood sampling.<br>• The recruitment lasted on average 10 minutes (more in the case of employees with poor language competency).<br>• About 40% of the employees have too little German language competency to make recruitment successful.<br>• The documentation tasks and questions from the interviewees result in considerable extra work.
2. How do employees react to the issue of high blood pressure?	• In principle, employees are interested.<br>• The topic is well-known.<br>• The topic is not taken seriously enough regarding themselves ('others have a problem, I don't; my blood pressure is always a little high, but that's normal' (2 answers).
3. How do employees react when they are approached about the study?	• At first they are usually interested; when they realize the effort involved, they often do not even listen properly.<br>• There is certain skepticism about a study thought to be conducted directly by the employer. A frequent question was: 'What does my employer need this data for?' These respondents are then also resistant to explanations regarding data protection (2 answers).
4. What is most likely to motivate employees to participate?	• Personal experience with the consequences of high blood pressure in the family or among friends.<br>• An existing blood pressure measurement device.<br>• Direct confrontation with the topic and a resulting concern for their own health.
5. Why do employees most often refuse to participate in the screening study?	• Data protection reasons.<br>• The effort involved in reading the study information.<br>• The lack of an own blood pressure measurement device and the unwillingness to purchase one for reasons of effort and cost.<br>• The time required for repeated measurements and documentation.
6. How could the recruitment process be improved?	• Through repeated measurements directly in the occupational health service, if necessary with the possibility of self-measurement so that no staff is involved. An unsolved problem is the resulting loss of working time.<br>• By lending measurement devices, e.g. against payment of a deposit.<br>• By translating the study information into other languages (Turkish, Greek, Italian).<br>• Through expanding recruitment in other Occupational Health Service locations of the company.<br>• Through initial measurements and correction in the departments<br>• Through material or financial incentives<br>• By organizing the addressees in groups to increase motivation and commitment<br>• By condensing the study information material and creating a more visually appealing design, e.g. in the form of a brochure.

Table S7. Occupational health physicians' hypertension prevention counseling –assessment and satisfaction of participants of intervention and control group.

	Mean (SD); median	
**Positive assessment of the counseling**	**IG (n=7)**	**CG (n=6)**
1. The counseling helped me to become clear about my health behavior	2.29 (0.95); 2.0	2.50 (1.05); 2.5
2. The expectations I had of the counseling were fulfilled	2.50 (1.22); 2.0	2.40 (1.14); 2.0
3. I would recommend the counseling to a friend/colleague	1.86 (1.21); 1.0	2.67 (1.37); 2.5
4. The counseling was an important trigger to my wish to change my health behavior	2.29 (1.25); 2.0	2.33 (1.50); 2.0
**Sum score (4 items)** [1)]	**2.14 (1.17); 1.7**	**2.38 (1.19); 2.1**

**Satisfaction…**	**IG (n=7)**	**CG (n=6)**
1. …with information received	1.86 (1.21); 1.0	2.33 (1.21); 2.5
2. …with the feedback and advice received	2.00 (1.41); 1.0	3.00 (1.26); 3.5
3. …with the way in which the OHP conducted the conversation	1.57 (1.13); 1.0	2.50 (1.05); 2.5
4. …with the way in which the OHP dealt with my personal situation	1.86 (1.21); 1.0	2.33 (1.03); 2.0
**Sum score (4 items)** [2)]	**1.82 (1.20); 1.2**	**2.54 (1.05); 2.7**

Legend: Possible values of Likert-scaled items from 1 'fully applicable to 5 'not applicable at all'.

Abbreviations:

CG= control group ('usual care' counseling for lifestyle changes); IG= intervention group (counseling for lifestyle changes by motivational interviewing); OHP= occupational health physician; SD= standard deviation.

[1)] $p_{(M\text{-}W)}$ = .534; r= 0.05

[2)] $p_{(M\text{-}W)}$ = .295; r= 0.03

Table S8. Satisfaction with the MI-method and respective procedures from the point of view of the occupational health physicians (n=4).

**Mean scores** [1)]	**Mean (SD); median (n=4)**
1. Satisfaction with the concept of motivational interviewing (MI; 6 items)	2.88 (0.16);2.9
2. Satisfaction with the introduction to become familiar with the study design (2 items)	2.38 (0.48);2.3
3. Satisfaction with the integration of MI into the daily work routine (2 items)	4.63 (0.25);4.5
4. Satisfaction with communication processes in the study team and the complex documentation procedure (2 items)	2.38 (0.48);2.3
**Single items**	**Mean (SD); median (n=4)**
**1 Satisfaction with MI- concept**	
1.1 The one-time MI-training was sufficient	2.75 (1.50);3.0
1.2 I am satisfied with my MI-qualification	2.50 (0.58);2.5
1.3 I am convinced of that the MI-concept is effective	2.25 (0.50);2.0
1.4 A supervision of users of the MI-concept in my opinion would be useful/necessary (*)	3.25 (1.50);3.0
1.5 Switching from usual care counseling to MI causes difficulties (*)	3.50 (1.29);3.5
1.6 I have adopted elements of MI in my daily consulting style	2.50 (1.29);2.5
**2 Satisfaction with integration of MI into the daily work routine**	
2.1 MI-counseling could be well-integrated into my daily work routines	2.25 (0.50);2.0
2.2 The documentation of the counseling sessions was too time-consuming (*)	4.50 (0.58);4.5
**3 Satisfaction with introduction into the study**	
3.1 I was well-informed about the study design at the beginning of my work on this study	2.00 (0.82);2.0
3.2 I would have wished for a more detailed introduction to the study design, study contents, and procedures (*)	4.25 (1.50);5.0
**4 Satisfaction with communication processes**	
4.1 I was satisfied with the communication within the study team	2.25 (0.50);2.0
4.2 I would (not) have liked to have more frequent information on the study status (*)	4.50 (1.00);5.0

Legend: Possible values of Likert-scaled mean scores from 1 'fully satisfied' to 6 'not satisfied at all'. Items ranging from 1 'fully applicable' to 6 'not applicable at all'.

[1)] Reversal of polarity of negatively formulated items; see (*); no. 1.4, 1.5, 2.2, 3.2, 4.2

Abbreviation: SD= standard deviation

Table S9. Experiences with the telephone MI-counseling by the health coach (intervention group.

	**Mean (SD); median (n=7)**
1. I was able to integrate the telephone counseling well into my daily routine	1.71 (0.49); 2.0
2. The atmosphere in the conversation was positive/pleasant	1.57 (0.53); 2.0
3. I felt understood and and that my views were taken seriously	1.43 (0.53); 1.0
4. I was listened to attentively during the conversation	1.14 (0.38); 1.0
5. I was encouraged to talk about my current health behavior	1.29 (0.49); 1.0
6. My decisions and achievements during the talks were acknowledged	1.14 (0.38); 1.0
7. I was able to achieve agreed goals at my own pace	1.14 (0.38); 1.0
8. The telephone counseling was an important trigger to want to change my health behavior	2.00 (0.58); 2.0
9. I would recommend the telephone conversations to a friend/colleague	1.29 (0.49); 1.0
10. The telephone counseling was helpful in staying on the ball with my health goals	1.29 (0.49); 1.0
11. The expectations I had of the telephone counseling have been fulfilled	1.57 (0.53); 2.0
12. Overall, I am very satisfied with the telephone counseling	1.57 (0.53); 2.0
**Sum score (12 items)**	**1.43 (0.33); 1.3**

Legend: Possible values of Likert-scaled items from 1 'not applicable at all' to 5 'fully applicable'.
Abbreviation: SD= standard deviation
